# Looking beyond Virus Detection in RNA Sequencing Data: Lessons Learned from a Community-Based Effort to Detect Cellular Plant Pathogens and Pests

**DOI:** 10.3390/plants12112139

**Published:** 2023-05-29

**Authors:** Annelies Haegeman, Yoika Foucart, Kris De Jonghe, Thomas Goedefroit, Maher Al Rwahnih, Neil Boonham, Thierry Candresse, Yahya Z. A. Gaafar, Oscar P. Hurtado-Gonzales, Zala Kogej Zwitter, Denis Kutnjak, Janja Lamovšek, Marie Lefebvre, Martha Malapi, Irena Mavrič Pleško, Serkan Önder, Jean-Sébastien Reynard, Ferran Salavert Pamblanco, Olivier Schumpp, Kristian Stevens, Chandan Pal, Lucie Tamisier, Çiğdem Ulubaş Serçe, Inge van Duivenbode, David W. Waite, Xiaojun Hu, Heiko Ziebell, Sébastien Massart

**Affiliations:** 1 Plant Sciences Unit, Flanders Research Institute for Agriculture, Fisheries and Food (ILVO), 9820 Merelbeke, Belgium; yoika.foucart@ilvo.vlaanderen.be (Y.F.); kris.dejonghe@ilvo.vlaanderen.be (K.D.J.); thomas.goedefroit@ilvo.vlaanderen.be (T.G.); 2 Foundation Plant Services, Department of Plant Pathology, University of California, Davis, CA 95616, USA; malrwahnih@ucdavis.edu (M.A.R.); kastevens@ucdavis.edu (K.S.); 3 School of Natural and Environmental Sciences, Newcastle University, Newcastle Upon Tyne NE1 7RU, UK; neil.boonham@newcastle.ac.uk (N.B.); f.salavert2@newcastle.ac.uk (F.S.P.); 4 UMR 1332 Biologie du Fruit et Pathologie, Institut National de Recherche pour l’Agriculture, l’Alimentation et l’Environnement (INRAE), Université de Bordeaux, 33882 Villenave-d’Ornon, France; thierry.candresse@inrae.fr (T.C.); marie.lefebvre@inrae.fr (M.L.); 5 Centre for Plant Health, Canadian Food Inspection Agency, 8801 East Saanich Road, North Saanich, BC V8L 1H3, Canada; yahya.gaafar@inspection.gc.ca; 6 Plant Germplasm Quarantine Program, Animal and Plant Health Inspection Service, United States Department of Agriculture (USDA-APHIS), Beltsville, ML 20705, USA; oscar.hurtado-gonzales@usda.gov (O.P.H.-G.); xiaojun.hu@usda.gov (X.H.); 7 Department of Biotechnology and Systems Biology, National Institute of Biology (NIB), 1000 Ljubljana, Slovenia; zala.kogej@nib.si (Z.K.Z.); denis.kutnjak@nib.si (D.K.); 8 Jožef Stefan International Postgraduate School, 1000 Ljubljana, Slovenia; 9 Plant Protection Department, Agricultural Institute of Slovenia (KIS), 1000 Ljubljana, Slovenia; janja.lamovsek@kis.si (J.L.); irena.mavricplesko@kis.si (I.M.P.); 10 Biotechnology Risk Analysis Program, Animal and Plant Health Inspection Service, United States Department of Agriculture (USDA-APHIS), Riverdale, ML 20737, USA; martha.malapi@usda.gov; 11 Department of Plant Protection, Faculty of Agriculture, Eskişehir Osmangazi University, Odunpazarı, Eskişehir 26160, Turkey; onderserkan@gmail.com; 12 Department of Plant Protection, Agroscope, 1260 Nyon, Switzerland; jean-sebastien.reynard@agroscope.admin.ch (J.-S.R.); olivier.schumpp@agroscope.admin.ch (O.S.); 13 Zespri International Limited, 400 Maunganui Road, Mount Maunganui 3116, New Zealand; chandan_pal143@yahoo.com; 14 Unités GAFL et Pathologie Végétale, Institut National de Recherche pour l’Agriculture, l’Alimentation et l’Environnement (INRAE), 84143 Montfavet, France; lucie.tamisier@inrae.fr; 15 Department of Plant Production and Technologies, Faculty of Agricultural Sciences and Technologies, Niğde Ömer Halisdemir University, 51240 Niğde, Turkey; culubas@gmail.com; 16 Dutch General Inspection Service for Agricultural Seed and Seed Potatoes (NAK), Randweg 14, 8304 AS Emmeloord, The Netherlands; i.vanduivenbode@nak.nl; 17 Plant Health and Environment Laboratory, Ministry for Primary Industries, Auckland 1140, New Zealand; david.waite@mpi.govt.nz; 18 Institute for Epidemiology and Pathogen Diagnostics, Federal Research Centre for Cultivated Plants, Julius Kühn Institute (JKI), Messeweg 11-12, 38104 Braunschweig, Germany; heiko.ziebell@julius-kuehn.de; 19 Plant Pathology Laboratory, University of Liège, Gembloux Agro-Bio Tech, TERRA, 5030 Gembloux, Belgium

**Keywords:** plant pathogen, diagnostics, high-throughput sequencing, metagenomics, metatranscriptomics, RNA-seq

## Abstract

High-throughput sequencing (HTS), more specifically RNA sequencing of plant tissues, has become an indispensable tool for plant virologists to detect and identify plant viruses. During the data analysis step, plant virologists typically compare the obtained sequences to reference virus databases. In this way, they are neglecting sequences without homologies to viruses, which usually represent the majority of sequencing reads. We hypothesized that traces of other pathogens might be detected in this unused sequence data. In the present study, our goal was to investigate whether total RNA-seq data, as generated for plant virus detection, is also suitable for the detection of other plant pathogens and pests. As proof of concept, we first analyzed RNA-seq datasets of plant materials with confirmed infections by cellular pathogens in order to check whether these non-viral pathogens could be easily detected in the data. Next, we set up a community effort to re-analyze existing Illumina RNA-seq datasets used for virus detection to check for the potential presence of non-viral pathogens or pests. In total, 101 datasets from 15 participants derived from 51 different plant species were re-analyzed, of which 37 were selected for subsequent in-depth analyses. In 29 of the 37 selected samples (78%), we found convincing traces of non-viral plant pathogens or pests. The organisms most frequently detected in this way were fungi (15/37 datasets), followed by insects (13/37) and mites (9/37). The presence of some of the detected pathogens was confirmed by independent (q)PCRs analyses. After communicating the results, 6 out of the 15 participants indicated that they were unaware of the possible presence of these pathogens in their sample(s). All participants indicated that they would broaden the scope of their bioinformatic analyses in future studies and thus check for the presence of non-viral pathogens. In conclusion, we show that it is possible to detect non-viral pathogens or pests from total RNA-seq datasets, in this case primarily fungi, insects, and mites. With this study, we hope to raise awareness among plant virologists that their data might be useful for fellow plant pathologists in other disciplines (mycology, entomology, bacteriology) as well.

## 1. Introduction

The concept of metagenomics, i.e., studying the genetic material of (environmental) organisms without the need to first culture or isolate them, was first introduced in 1998 [1], and its application in research grew exponentially with the introduction of high-throughput sequencing (HTS) in 2005, when the sequencing of millions of DNA molecules in parallel at a relatively low cost became available. Many different applications of metagenomics exist, and, not surprisingly, its potential for diagnostic applications increased tremendously [2,3], especially when traditional targeted diagnostic tests fail. Metagenomics relies on the random sequencing of (in some cases sheared) extracted nucleic acids using HTS technologies, allowing the investigation of the taxonomic composition of nucleic acids of mixed origin. Notably, HTS technologies can also be applied for taxonomic purpose using a second strategy, called metabarcoding, that relies on the amplification by PCR of a small genomic region (typically with limited taxonomic information) of the targeted species prior to HTS [4].

Using metagenomics, all genetic material in a sample is analyzed; in traditional targeted molecular testing, only molecules specific for the pathogen are targeted. This makes metagenomics very much suited for difficult cases of infections with uncommon, unexpected, or new pathogens. HTS can deliver a full qualitative and quantitative analysis of the DNA or RNA sequences within a sample in a single test, and thereby promises next level diagnostics [5]. Clinical application of metagenomics can not only identify pathogens but can also yield additional genomic information of the pathogen that can, for example, be used to select an effective antibiotic or antiviral treatment [6].

In plant pathology, the potential of HTS for virus detection was recognized early by some pioneering studies [7,8,9,10], and, further on, several different protocols have been developed, each with their own benefits and drawbacks [11]. Small RNA sequencing can be used to detect viruses via the small RNAs produced by the plant RNAi defense machinery [7]. Other methods were introduced to enrich viral RNA in the extract. such as dsRNA sequencing and VANA (Virion-Associated Nucleic Acid) sequencing. Total RNA sequencing (RNA-seq) has recently become increasingly popular because of its ease of use and broad detection spectrum, although it has the drawback that more sequencing reads are needed to detect viral RNA against the background of plant RNA [12]. To increase RNA-seq sensitivity, plant ribosomal RNA (rRNA) is often removed prior to library preparation, which can lead to a 10-fold enrichment of viral RNA [13]. This RNA-seq approach is now relatively common among plant virologists to detect and identify viruses in research and, progressively, also in official diagnostic applications such as post-entry quarantine or certification [4,14].

As in metagenomics applications, RNA-sequencing of plant materials will not only include viral RNA sequences, but also RNA derived from other microorganisms living on the plant sample surface (phyllosphere) or in the endosphere of the plant, as well as environmental RNA (from, for example, insects physically associated with the sample). Mining the HTS data can give a complete overview of the organisms present in/on the sample, among which are potentially pathogenic agents. However, for several reasons, the technique has never really been used for true untargeted detection of non-viral plant pathogens (reviewed by [15]). The interest in metagenomics as a diagnostic tool has gained popularity in the last few years with the rise of long read sequencing techniques [16,17]. Alternatively, specific sequences or e-probes can be used to mine metagenomics and RNA-seq datasets for the presence of certain pathogens [18,19]. Finally, mitochondrial metagenomics, where samples are enriched in mitochondrial fragments prior to library preparation, also seems promising when sufficient data is present in reference databases [20].

As explained above, RNA-seq has become an essential tool for the detection and identification of plant viruses but has received little attention for the diagnosis of other pathogens and pests. Consequently, plant virologists generate numerous RNA-seq datasets but typically only investigate these datasets for the presence of viruses, being unaware of the possible presence of other plant pathogens or pests. In this study, we hypothesized that RNA-seq data generated by virologists could be used to detect non-viral pathogens and pests as well. To check this hypothesis, we first analyzed RNA-seq data of plant materials known to be infected by cellular pathogens to see if these pathogens could be detected and correctly identified from the sequencing data. This was performed using both publicly available datasets and newly generated data. Next, we organized an “RNA-seq community effort”, in which existing RNA-seq datasets from different diagnostic laboratories generated for the detection of viruses were revisited and checked for the potential presence of non-viral plant pathogens and pests. Participants were actively involved in the re-analysis of their data, and were asked to give feedback on the results, including the experimental confirmation of the presence of the detected cellular pathogens by (q)PCR. The goal of this effort was two-fold: (1) to provide a broad vision of the type of pathogens that are typically detected in these RNA-seq datasets as well as on the relative proportion of rRNA and mRNA derived from each organism group (host plant, viruses, bacteria, phytoplasmas, fungi, insects, mites) in a broad range of plant materials and (2) to raise awareness among plant virologists that their data might be useful for other plant pathologists as well, which hopefully will stimulate collaboration with bacteriologists, mycologists, and entomologists.

## 2. Results

### 2.1. Various Non-Viral Pathogens Can Be Detected and Identified in Plant RNA-Seq Data

As proof of principle, 22 total RNA-seq datasets derived from plant materials with known cellular pathogen infections were analyzed to see whether or not these pathogens could be detected in the RNA-seq data. These datasets can be regarded as reference samples (positive controls), since they were obtained from plants with confirmed infections. An overview of the datasets, of the detected pathogens, and of the observed number of reads per million (rpm) (defined as the number of pathogen reads (pairs, in the case of paired-end sequencing) per million of total reads) is provided in Table 1.

The results show that, for 19 of the 22 datasets, we were able to detect the pathogen in the RNA-seq data with more than 10 rpm. The relative abundance of the pathogen varies greatly (in this case, from 1 to 51,974 rpm) depending on the pathogen and the host tissue. In some cases, the numbers are extremely high, for example *Plasmopara viticola* in grapevine leaves has 51,974 rpm assigned, corresponding to >5.2% of the total number of reads. In other cases, for example, for *Xylella fastidiosa* in willow leaves, the observed numbers are very low (35 rpm); thus, the sensitivity of detection can be limited.

Another observation that can be made is that pathogen species assignment by Kraken2 is sometimes unclear and can even be incorrect (see, for example, *Puccinia horiana,* which was reported as *P. graminis* or *A. solani*, which is most likely not the *Alternaria* species occurring in carrot). This is well illustrated in samples KIS_V3408 and KIS_V3408dup, which are independent samples from the same plant processed in parallel. Both samples showed a lot of reads attributed to the *Phytophthora* genus, but the species assignment was unclear in one of the samples.

**Figure 1:**
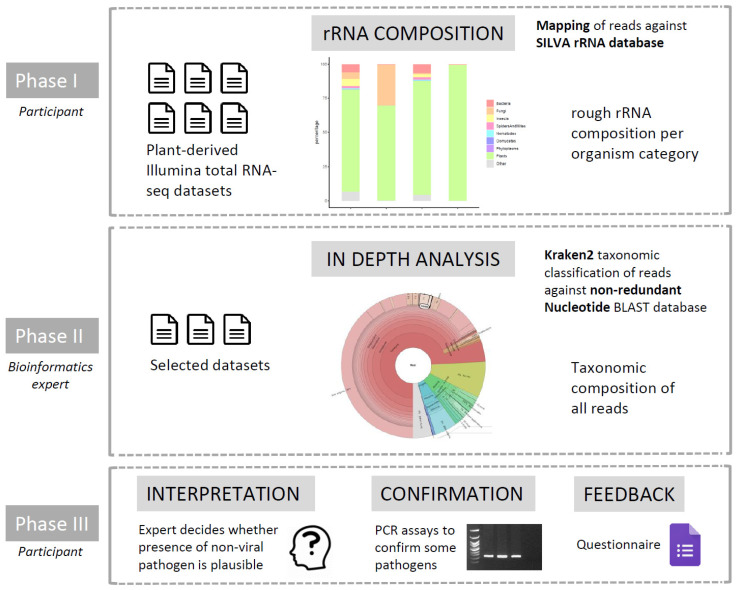
Overview of the different phases in the “RNA-seq community effort”, in which total RNA-seq datasets previously analyzed for the presence of viruses were re-analyzed in order to find possible traces of non-viral pathogens and pests.

After confirming that it is indeed possible to detect non-viral pathogens in total RNA-seq datasets, we set up an “RNA-seq community effort”, with the goal to re-analyze existing RNA-seq datasets available in the plant virology community for the presence of other—possibly unnoticed—pathogens (Figure 1). This community effort consisted of three phases. In Phase I, the participants analyzed the data themselves by mapping the generated reads against the SILVA ribosomal RNA database to obtain a first evaluation of the fraction of non-plant reads that might be present in the data. SILVA provides comprehensive, quality checked, and regularly updated datasets of aligned small (16S/18S, SSU) and large subunit (23S/28S, LSU) ribosomal RNA (rRNA) sequences for all three domains of life (*Bacteria*, *Archaea*, and *Eukarya*). In Phase II, performed by a bioinformatics expert, some selected datasets (showing a substantial amount of non-plant rRNA reads) were selected for a more thorough taxonomic classification of all RNA reads (instead of only the rRNA reads) using Kraken2. Finally, in Phase III, the presence of pathogens and pests detected in Phase II was evaluated by participants and, in some cases, subjected to experimental (q)PCR confirmatory efforts. Finally, participants answered a survey designed to evaluate the usefulness of this approach to detect pathogens and pests.

### 2.2. RNA-Seq Community Effort PHASE I

#### 2.2.1. A Total of 101 RNA-Seq Datasets Were Re-Analyzed by 15 Participants

An overview of all datasets that were entered in the “RNA-seq community effort” can be found in File S1. In total, 15 participants from 10 countries participated. One participant worked exclusively in a diagnostic lab, while 13 were from labs doing both research and diagnostics, and 1 was from a research-only lab. In total, 101 datasets were analyzed, with individual participants providing between 1 and 20 datasets. An overview of the datasets per participant is shown in Figure S1. The datasets were derived from 51 different plant species belonging to 29 different families. Most species were food crops (74), but there were also some industrial (5) and ornamental crops (11), as well as some wild species (11). The most frequent species was grapevine (10 datasets), followed by potato (6) and several fruits, such as apple (5), blackberry (4), and pear (4), as well as rose (4) and tomato (4). Four samples represented pools of different plant species. The vast majority of the datasets were derived from leaf material (69), branches (1), petioles (10), or combined leaves + petioles/branches (9), while only a minority of them were derived from other plant parts, such as tubers/rootstocks (10) or pollen (2). At least 9 different RNA extraction methods/kits were used (File S1). About half of the samples (45) were treated with DNase during RNA extraction, and the majority of the samples underwent an rRNA depletion step (70) prior to HTS. Interestingly, one participant entered the same sample twice, once processed with and once without an rRNA depletion step. Most samples were paired-end sequenced (70), with 2 × 150 bp being the most popular read length (40), while, for the single-end datasets, 1 × 75 bp occurred the most often (25). The number of reads ranged from 1.2 M to 142.0 M per dataset, with an average of 22 M.

#### 2.2.2. rRNA Depletion during Library Prep Reduced the Amount of Plant rRNA with Very Variable Efficiencies

**Figure 2:**
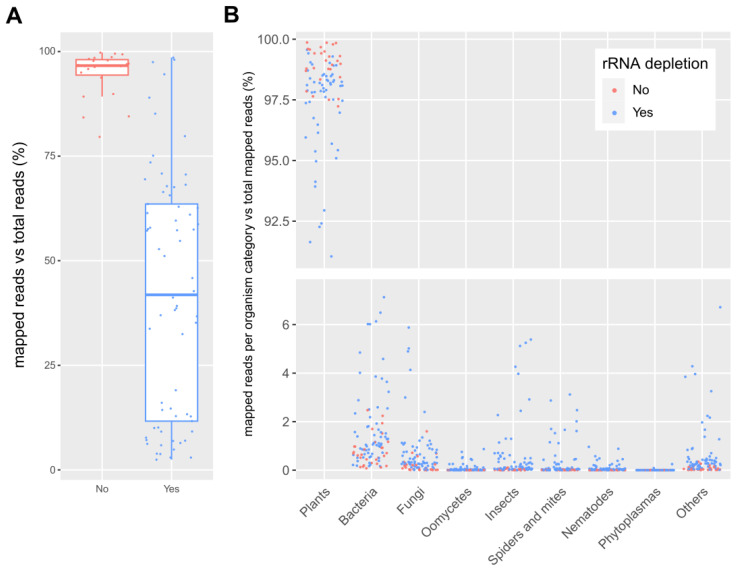
(**A**): Boxplot of the percentage of reads mapped against the SILVA LSU rRNA database compared to the total number of reads in the dataset for samples with (“Yes”) and without (“No”) an rRNA depletion step. (**B**): Percentage of reads mapped against the SILVA LSU rRNA database per organism category relative to the total number of mapped reads. Each dot represents one dataset, and colors indicate if rRNA depletion was applied during library preparation. Some outliers are not visible on the graph.

The participants mapped their datasets against the SILVA Large Subunit (LSU) database and reported the number of mapped reads per database entry. Since each participant independently performed the mapping, data processing steps differed slightly between them (File S1). Most datasets underwent some basic preprocessing in the form of quality filtering (99) and/or adapter removal (92), in some cases followed by merging of forward and reverse reads (35) and/or duplicate removal (12). Of the 101 datasets, 79 were mapped using BWA, 18 using Geneious, and 4 using CLC Genomics Workbench. The percentage of reads that mapped against the rRNA database is shown in Figure 2A. On average, 95 ± 5% (standard deviation) of the reads mapped in case no rRNA depletion step was performed, while only 42 ± 29% of the reads mapped in case an rRNA depletion step was included during library preparation. This difference proves that rRNA removal kits are useful to boost the number of non rRNA reads, although results are very variable (Figure 2A). This was also well reflected in the sample from which two datasets were available, one prepared with and one prepared without an rRNA depletion step. In the dataset derived from the library without rRNA depletion, 94% of the reads mapped to the rRNA database, while this was only 3% in the case of the dataset derived from the library with rRNA depletion.

The sequences that mapped against the rRNA database were assigned to broad categories of organisms in order to study the global composition of the rRNA of all samples. An overview of this rRNA composition is shown in Figure 2B. The vast majority of the mapped rRNA reads belonged to the plants category (on average, 94.53 ± 1.03%), followed by bacteria (on average, 2.33 ± 0.53%), fungi (on average, 1.57 ± 0.58%), insects (on average, 0.66 ± 0.22%) and spiders and mites (on average, 0.24 ± 0.06%). In some datasets, the rRNA levels for bacteria, fungi, or insects reached levels higher than 3% of the total reads. Other categories (phytoplasmas, oomycetes, and nematodes) all had an average percentage of mapped reads below 0.1%.

### 2.3. RNA-Seq Community Effort Phase II

#### 2.3.1. The Reads from the RNA-Seq Datasets Analyzed in Detail in Phase II Are Primarily Assigned to Fungi and Bacteria, but also to Insects and Mites

**Figure 3:**
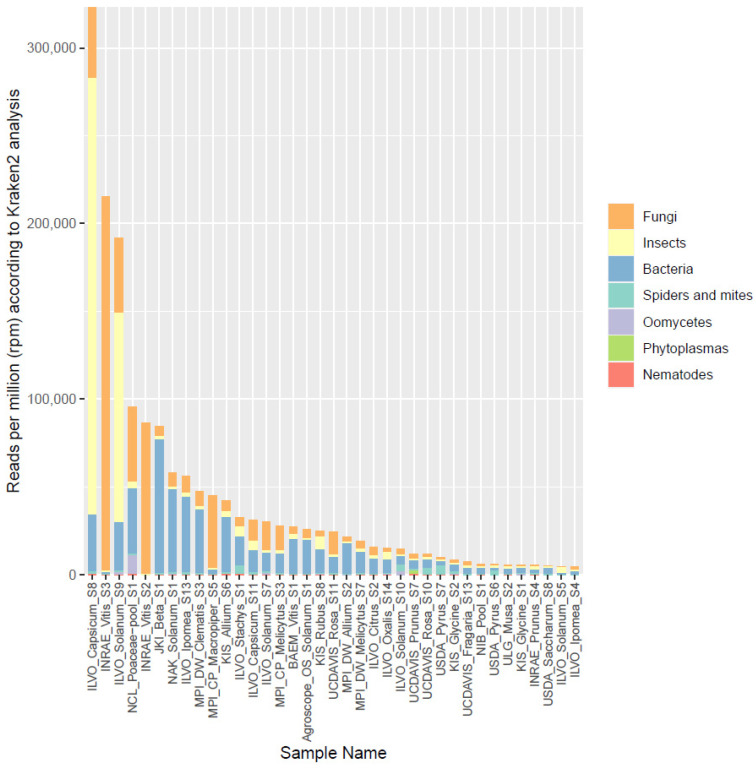
Overview of the proportion of reads (in reads per million) assigned by Kraken2 to different broad organismal categories (fungi, insects, bacteria, spiders and mites, oomycetes, phytoplasmas, and nematodes) for the 37 selected datasets.

Based on the results of Phase I, 37 of the 101 datasets were selected to be analyzed in more detail by classifying the reads taxonomically using Kraken2, a k-mer based lowest common ancestor method. Resulting Krona plots showing the taxonomic diversity resulting from these annotation steps are available for all datasets on https://gitlab.com/ilvo/PHBN-WP4-RNAseq_Community_Screening. Figure 3 shows the relative number of reads (in rpm) that were assigned to the following organism categories: fungi, insects, bacteria, spiders and mites, oomycetes, phytoplasmas, and nematodes. The total number of rpm assigned to one of these categories ranged from 4549 to 322,901 rpm, corresponding to 0.45–32% of the reads, with a median of 2.4%. The reads assigned to plants ranged from 30 to 92% (File S1). Similar to the results of Phase I, fungi and bacteria were the categories showing the highest representation, while phytoplasmas and oomycetes were detected only in some samples and at relatively low levels. Nematode sequences seemed to be present as low-level background in almost all samples. Surprisingly, in almost all samples, insect or mite reads were also found, even up to 25% of the total number of reads for one sample. When these results were compared to those from Phase I, a weak but significant correlation was found between the fraction of non-plant reads identified during the rRNA mapping and the level of non-plant reads identified in the Kraken2 analysis (Pearson correlation coefficient (PCC) = 0.38, *p* = 0.021). When the same was performed separately for each broad organismal category, a significant correlation (*p* < 0.05) was found for phytoplasmas (PCC = 0.94, *p* < 2.2 × 10^−16^, insects (PCC = 0.90, *p* = 2.982 × 10^−14^), fungi (PCC = 0.76, *p* = 3.558 × 10^−8^), and spiders and mites (PCC = 0.34, *p* = 0.038). After the removal of one outlier, the bacteria group also showed a significant correlation (PCC = 0.45, *p* = 0.006). Only the nematode and oomycete categories did not show significant correlations between Phase I and Phase II results, probably due to the low number of reads attributed to these categories in both analyses.

#### 2.3.2. Several Non-Viral Pathogens/Pests Were Detected, Mainly Fungi and Insects

The Krona plots were carefully examined by participants for the presence of reads assigned to (non-viral) pathogens. Based on the feedback from participants on pathogens that might be present in the datasets, we set a global relative occurrence threshold of at least 100 rpm (i.e., >0.01%) in the Kraken2 analyses to consider a pathogen or pest to be present. This threshold was deliberately set relatively high to avoid false positives. Indeed, in our previous analysis of datasets derived from leaves with known infections (see Section 2.1 and Table 1), we noticed that several pathogens were observed at lower abundances. Hence, by using the threshold of 100 rpm, we can expect some false negative results, i.e., unnoticed pathogens. Nevertheless, we decided to continue with this relatively conservative threshold in order to avoid false positives. An overview of the datasets in which pathogens were thus identified is shown in Table 2.

**Figure 4:**
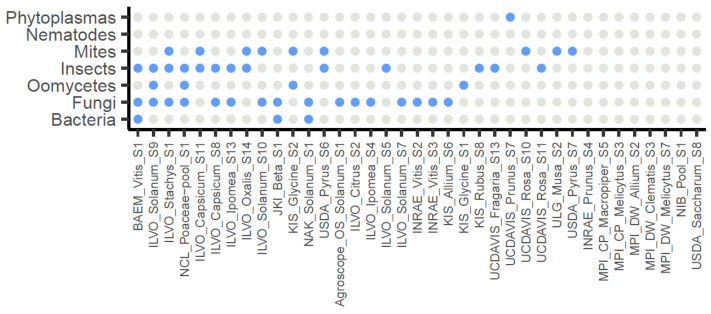
Overview of the 37 selected datasets and the potential presence of non-viral pathogens and pests as grouped by broad organismal categories. Grey: no observed presence (<100 rpm), blue: observed presence (≥100 rpm).

Considering the 37 selected datasets, in total 67 pathogens (39 different taxa) were detected (≥100 rpm) from 29 of the datasets (78%), with an abundance ranging from 100 to 107,450 rpm. Fungal pathogens were the most abundant, with 31 detections (in 16/37 datasets, 43%), with the most common taxa being *Alternaria* sp. (found in eight datasets), *Botrytis cinerea* (found in seven datasets), and *Fusarium sp.* (found in five datasets). Insect pests were detected 19 times (in 13/37 datasets, 35%): 9 aphids (3 different species), 5 thrips, 4 fruit flies, and 1 mealybug. Mites were detected 9 times (in 9/37 datasets, 24%), followed by oomycetes (in 4/37 datasets, 11%), plant pathogenic bacteria (in 3/37 datasets, 8%), and phytoplasma (in 1/37 dataset, 3%) (Figure 4).

### 2.4. RNA-Seq Community Effort Phase III

#### 2.4.1. About Half of the Participants Were Unaware of the Presence of Reads from Non-Viral Pathogens in Their Dataset(s)

The participants completed a questionnaire (File S2) that asked their opinion about the usefulness of and previous experience(s) with these analyses. The questionnaire answers (File S3) revealed that only 6/15 participants were aware that their sample had indications of the presence of non-viral pathogen(s), while, for 11/15 participants, at least one non-viral pathogen was detected in at least one of their datasets. A total of 14/15 participants were aware of the existence of metagenomics techniques applicable to RNA-seq datasets, but only 6/15 had previously used similar approaches to taxonomically classify all reads or contigs against databases that contained cellular organisms and not only viruses. These were almost exclusively participants with an expert bioinformatics level. Most participants also compared the viruses found with their previous analyses and found a good correspondence, although five participants identified more viruses than previously identified by their own pipeline. Regardless of their proficiency in bioinformatic analyses, all participants indicated that they will probably use these metagenomics methods in the future.

#### 2.4.2. (q)PCR Confirmation of Pathogen Detection

Since a relatively high number of pathogens were found, we decided to try to confirm some of these presumed infections. To have a representative view on different organisms, a bacterium (*Pectobacterium* sp.), a phytoplasma (‘*Ca.* Phytoplasma pruni’), and two fungi (*Botrytis cinerea* and *Alternaria* sp.) were selected and tested by (q)PCR on DNA extracted from the same biological material from which the RNA-seq data was derived. Since the two fungi were observed in multiple samples (Table 2), we selected a few samples with variable read numbers. Results are shown in Table 3. Both the presence of the bacterium and the phytoplasma were confirmed by the independent assays. Interestingly, for *Botrytis cinerea*, the samples where a very high rpm was observed in the Kraken2 analyses were PCR positive, but when the abundance was lower (≤131 rpm), the presence could not be confirmed. This can have several reasons, one being that the reads may have been assigned to the wrong species (false positive of Kraken2 analysis); another reason could be that the fungus may have been present at a level below the PCR assay detection limit (false negative of PCR). For *Alternaria* sp., the threshold at which the fungus could still be detected was lower; a sample with 30 rpm still tested positive by PCR, while another sample, with only 17 rpm, tested negative.

## 3. Discussion

The identification and monitoring of pathogens and pests are crucial to the health and productivity of agricultural systems. Several methods, from microscopic observation over Enzyme Linked Immunosorbent Assay (ELISA) to nucleic acid-based techniques, e.g., PCR, have been developed over time to improve the detection of plant pathogens and pests. In recent years, the advent of HTS technologies has provided a promising new approach for the detection of plant pathogens and pests in an untargeted way. One of the most frequent applications of HTS technologies in plant pest diagnostics is the identification of viral or cellular pathogens causing novel diseases or diseases of unknown etiology [4]. HTS technologies are already applied in certification schemes, post-quarantine testing, disease surveillance, and outbreak tracing, and the generated data can be the basis for, e.g., designing primers with improved analytical specificity and inclusiveness [21].

In plant virology, shotgun-based protocols have become the standard for complete virome scanning or for diagnosing difficult samples in case targeted tests fail. Although different types of library preparations exist [22], one of the more popular methods is total RNA sequencing because it allows researchers to detect RNA viruses, DNA viruses, and viroids present in a sample. RNA is extracted from the plant tissue, plant rRNA is optionally depleted using specific probes, and the remaining RNA sequenced. To detect viruses, the resulting reads (or contigs, if an assembly is performed) are subsequently compared to a virus reference database [23]. In other plant pathology disciplines, this method is much less popular since the more traditional detection methods are often quicker and cheaper. Indeed, Tedersoo et al. [15] observed that metagenomics and RNA-seq studies specifically targeting non-viral plant pathogens are rare. Nevertheless, RNA-seq had already been used in 2015, for example, to identify not only viruses but also bacterial and fungal pathogens in mosquitoes [24]. In plants, most RNA-seq studies are investigating the plants’ responses after pathogen infection and/or the expression of pathogen effectors [25,26,27] rather than trying to detect pathogens. Recently, however, RNA-seq from the rhizosphere or phyllosphere has been used to study the complex ecological interactions between plants and plant-associated (micro-)organisms [28,29]. Moreover, nanopore sequencing using the handheld MinION sequencer has seen renewed interest in disease detection using metagenomics or RNA-seq, and it has been successfully used to detect fungi and bacteria, sometimes with identification down to the strain level [30,31,32,33,34]. In addition, (meta)genomics is being progressively adopted for the efficient tracing of the origin(s) of outbreaks or for high resolution phylogeographic studies of pathogens, such as *Xylella fastidiosa* [35] or *Fusarium graminearum* [36].

In the present study, we demonstrated that it is indeed possible to detect and identify reads from cellular organisms in datasets prepared for plant virus detection. In all datasets examined with confirmed pathogen infections, reads from the pathogen could be retrieved. In total, 101 datasets without information on the possible presence of cellular organisms were also analyzed by mapping the reads against an rRNA database. In this way, by determining the number of reads presumably derived from rRNA, we could evaluate the effectiveness of the ribodepletion step. Including an rRNA depletion step was generally able to greatly decrease the rRNA fraction of the dataset, from an average of 95 to 42%. However, its efficiency proved highly variable across datasets. This variable efficiency could be due to various factors, such as the biological material (different tissues, different plant species), the use of different kits, or the experience of the laboratories. The (remaining) rRNA fraction also showed that bacteria and fungi were among the most prevalent non-plant sequences. Thirty-seven datasets that showed a substantial amount of non-plant rRNA were selected for more detailed analysis using Kraken2. Overall, the number of non-plant sequences (especially fungal and insect ones) correlated well with the number of mapped rRNA sequences against the SILVA database, making the latter method suitable for a fast first analysis on the (r)RNA composition. The amount of reads that were not assigned to plants varied greatly between the 37 selected datasets, ranging from 1 to >30% of the total number of reads. In general, bacteria were present in all samples at a relatively stable fraction, presumably representing the plants’ endophytic microbiome. Fungi were also represented in all samples, but, in some samples, they reached a relatively high abundance. The same was true for insects (Figure 3). When trying to identify plant pathogens, plant pathogenic bacteria were detected much less frequently than fungi. Indeed, they were found in only 4 cases (of which 1 was phytoplasma), while plant pathogenic fungi were detected in 31 of the 37 datasets. Nevertheless, some bacterial pathogens were detected at high abundance, such as ‘*Ca*. Phytoplasma pruni.’ and *Pantoea agglomerans*, with 8815 and 12,325 rpm, respectively. On the other hand, for *Pectobacterium* sp., for example, a relatively low abundance of pathogen reads among all sequencing reads was observed (145 rpm). Nevertheless, that level was high enough to lead to a positive PCR diagnostic, triggering retrospective phytosanitary measures for this sample. In the case of fungal pathogens, we observed very high abundances of pathogen reads (up to 84,898 rpm) in some cases, of which some were also confirmed by PCR (Table 3). Nevertheless, detection at low abundance levels could also be confirmed by PCR, as low as 30 rpm for *Alternaria* sp., for example. However, *Botrytis cinerea* could not be confirmed in a sample in which it was observed at 131 rpm. This variability in relative abundance of reads derived from the pathogen is most likely dependent on the specific sample, pathogen species, and detection assays used. Globally, 29 of the 37 selected datasets (78%) showed evidence of plant pathogens or pests, which were mostly fungi, insects, or mites (Figure 4). The latter categories were rather surprising, since the presence of pests were, in many cases, not expected and were not thought to leave so many traces of RNA in and on plant tissues. This is especially interesting for virologists, since many viruses are transmitted by insects or mites. Using the same RNA-seq datasets they use for virus detection, virologists could thus potentially look for a vector, or even use the dataset to identify a vector candidate in case none is known yet.

Even though it is clear that pathogen and pest reads can be identified in the RNA-seq datasets, the relative number of reads derived from the pathogen (compared to the total amount of reads) was sometimes very low in samples with validated infections. Several factors can explain low pathogen read numbers and inter-sample variability. There can be technical reasons that limit the chance of detecting pathogen reads, for example, the application and/or efficiency of a ribodepletion step as described above, or a limited number of reads generated. Raising the sequencing depth might be a solution to generate more pathogen reads. However, this can be limited by the cross-contamination background between samples, as this was previously observed to lead to inconclusive results when validating HTS technologies for virus indexing [37]. Next to technical reasons, there can also be biological reasons. First of all, most viruses infecting plants tend to have a systemic distribution, although this depends on the host plant and season. The situation for cellular pathogens is different, as some of them are restricted to specific tissues. Therefore, if the pathogen does not primarily reside in the sampled tissue, the chances for its detection are low. This was the case, for example, for *Xylella fastidiosa*, ‘*Ca.* Liberibacter sp.’, some ‘*Ca.* Phytoplasma sp.’, *Erwinia amylovora*, and *Xanthomonas campestris* in leaves, which were observed in sometimes very low read numbers. The second factor is the temporal dynamics of the pathogen in the host, more particularly during the latent phase when its concentration can be very low. For example, reads from *Erwinia amylovora* and *Xanthomonas campestris* were detected only at a low frequency in both leaf and root tissues (<10 rpm). Both pathogens are known to infect specific cell types and different tissues in different stages of their life cycle [38,39], hence the time of sampling since first infection can have an influence on the number of reads observed. On the contrary, when the sampled tissue presents primary symptoms of infection, the concentration of the same pathogen can be very high, as observed, for example, for *Verticillium dahliae* (37,732 rpm). Finally, the bacterial microbiome always corresponds to the most abundant non-plant sequences in the samples. This confirms that a complex community of microorganisms is present inside or at the surface of plant tissues. The composition and diversity of this community can impact the plant pathogen by limiting its development [40,41] and therefore reducing the proportion of reads in the dataset despite its presence.

A last observation was that the species assignment by Kraken2 was not always correct. Technical reasons might cause these misassignments, for example, the algorithm used or the incompleteness, biases, or mistakes in the used sequence database. This was also observed by Yang et al. [34], who were only able to identify a boxwood blight pathogen in metagenomic data after customizing the database. On the other hand, sequence information in nucleotide databases may be biased towards pathogens, possibly leading to wrong conclusions about a pathogen presence when only a non-pathogenic relative, for which there is no sequence information in the database, is present. Indeed, metagenomic classifiers tend to perform very well when closely related sequences are present in the database, but less so in the case that this information is lacking [42]. This phenomenon can be reinforced by a low number of generated reads, which limit the coverage and representation of the pathogen genome.

The preliminary results of this study have shown the potential of metatranscriptomics as a universal plant pathogen and pest detection method, allowing the detection of all expected pathogens from samples with confirmed infections, although sometimes as a very small proportion of sequencing reads. Nevertheless, RNA-seq as a stand-alone test for the detection of cellular pathogens is not likely to become a standard diagnostic tool due to some of the drawbacks discussed above. Moreover, it presents other drawbacks, such as the cost and complexity of library preparation, the huge number of reads generated (despite the continuous reduction of sequencing prices), the complex bioinformatics infrastructure needed, and the expected longer turnaround time of this method as compared to targeted methods. Some of these drawbacks could be potentially alleviated by the development of quick(er) sequencing platforms and the availability of easy-to-use data analysis platforms such as CZID (formerly IDseq) [43] and Kaiju [44].

In conclusion, we recommend that plant virologists generating RNA-seq data should also process their data using a metagenomic classifier in order to have an idea of the global composition of the sample and the eventual presence of cellular pathogens. A questionnaire that was distributed across the virologists that participated in this study revealed that all of them planned to use the technique in the future. This may stimulate collaboration and communication between plant virologists and bacteriologists, mycologists, and entomologists. Rather than working with fixed thresholds to decide whether or not a pathogen is present, we recommend the use of proper controls, such as spiking the sample with genetic material from a known pathogen as a positive control or including negative controls to obtain an idea on the cross-over contamination (see also [4]). Nevertheless, in case of finding a pathogen, there should always be an interpretation by the plant pathologist to check if the detected pathogen species matches the host plant and geographical origin, and an independent confirmation assay is recommended.

## 4. Materials and Methods

### 4.1. Analysis of Published and Newly Generated Plant-Derived RNA-Seq Data for Expected Pathogens

We first checked whether different types of non-viral plant pathogens could be detected and identified from plant total RNA-seq data, a method commonly used to detect plant viruses. The datasets used were selected in order to cover a broad range of pathogens (bacteria, fungi, oomycetes) and host plant tissues (leaves, fruits, phloem, flower buds). To do this, we downloaded 13 publicly available datasets from NCBI’s Sequence Read Archive (SRA). These datasets were plant-derived RNA-seq datasets with confirmed infection of a plant pathogen. We selected datasets with the following pathogens: the vascular bacteria ‘*Candidatus* Liberibacter sp.’ (SRR5100668: potato leaves, SRR10148792: orange leaves, SRR8295844: orange phloem) and ‘*Candidatus* Phytoplasma sp.’ (SRR9225242: tomato leaves, SRR7186379: soybean flower bud, SRR17253894: Chinese cherry fruit); the fungus *Verticillium dahliae* (SRR8003868: sticky nightshade roots, SRR6760520: olive roots, SRR6053344: upland cotton roots, SRR1525437: olive leaves); the bacterium *Erwinia amylovora* (SRR7814393: apple leaves); and the bacterium *Xanthomonas* sp. (SRR13488408: pepper leaves, ERR2036424: wheat roots). In addition to the downloaded datasets, we also generated 9 new RNA-seq datasets from plants with known and confirmed infections from different types of pathogens (bacteria, phytoplasmas, fungi, and oomycetes). Leaves were collected from *Xylella fastidiosa* infected goat willow; ‘*Candidatus* Phytoplasma sp.’ infected carrot and faba bean; *Puccinia horiana* infected *Chrysanthemum* sp.; *Alternaria* sp. infected *Daucus carota* and potato; and *Phytophthora infestans* and tomato and grapevine infected with an unidentified downy mildew. RNA was extracted using the RNeasy Plant Mini kit (Qiagen, Venlo, The Netherlands) and DNase treated following the manufacturer’s recommendations (ThermoFisher Scientific, Antwerp, Belgium). Ribosomal RNAs were removed using a RiboMinus Plant Kit for RNA-Seq (ThermoFisher Scientific) before cDNA library synthesis with the Illumina TruSeq Stranded RNA library prep kit (Illumina Inc., San Diego, CA, USA) and sequenced on an Illumina NextSeq500 (2 × 150 nt) in a multiplexed format (GIGA-Genomics Facility, Université de Liège, Liège, Belgium). Raw data was submitted to SRA under BioProject number PRJNA961939. Data from both the SRA-derived datasets, as well as the newly generated datasets, were further processed as follows. Adapters were removed from the sequencing data using cutadapt [45]. Next, the reads were classified taxonomically with the k-mer based Kraken2 software [46], using the lowest common ancestor method (LCA) for assignments at different taxonomic levels. For the database for the Kraken2 analysis, a local version of the complete Genbank non-redundant Nucleotide database converted to a Kraken2 index was used [47]. The results were visualized using Krona [48], and the rpm of the expected pathogens was calculated. The taxonomic resolution at which Kraken2 classified the reads was evaluated by visually checking if the reads from the pathogen genus almost all matched to the same species (=species level resolution), or to multiple species with similar read numbers. In the latter case, we decided to report the taxon at the genus level. Moreover, in case of a low relative occurrence of the species (<20 rpm), the taxon was reported at the genus level to avoid species assignments supported by a small number of reads.

### 4.2. RNA-Seq Community Effort

Next, we organized an “RNA-seq community effort” among plant virologists in three phases in order to evaluate the possible presence of non-viral pathogens and pests in some of their previously generated datasets. In the first phase, called “prescreening”, participants mapped their data against an rRNA database. This was used as a very rough but easy method to let participants prescreen their own samples for potential presence of non-plant rRNA. In the second phase, referred to as “in depth analysis”, a selection of datasets from each participant were transferred to one lab (ILVO) for a detailed taxonomic analysis by a bioinformatics expert. In the third phase, called “feedback and confirmation”, results of the detailed analysis were shared with participants for interpretation and further feedback, and the presence of some pathogens was experimentally checked. A general overview of our “RNA-seq community effort” is shown in Figure 1, and detailed information on the different phases is explained below.

#### 4.2.1. Phase I: Prescreening

Since we wanted to involve the participants as much as possible with the data analysis, we opted for a prescreening phase that participants could perform in-house. The goal of the prescreening phase was to obtain a general overview of what types of organisms are present in the datasets in order to make a selection of samples interesting for more detailed analysis. This prescreening phase needed to be relatively fast and easily accessible in order to allow participants with minimal experience in bioinformatics to participate as well. Hence, we opted for a mapping approach because mapping is a basic bioinformatics method used in virtually every lab dealing with HTS data, and it is relatively fast and therefore capable of processing many different samples. Participants were allowed to apply their mapping algorithm of choice on as many of their samples as they wanted. The only prerequisite was that the samples were plant-derived total RNA (no small RNAs) and sequenced with Illumina technology. For the mapping database, we opted for the SILVA LSU rRNA database (v.132) [49] (“SILVA_132_LSURef_tax_silva_trunc.fasta” as downloaded from the ARB-SILVA archive: https://ftp.arb-silva.de/release_132/Exports/, accessed on 26 March 2020). This database was chosen because (1) rRNA is typically very abundant in RNA-seq data (even when rRNA depletion was performed during library preparation); (2) it has a large sequence diversity across different kingdoms; (3) it is curated and non-redundant; and (4) it is relatively small and, hence, easy to distribute. The mapping database was slightly modified to include organism categories (bacteria, fungi, insects, spiders and mites, plants, nematodes, oomycetes, phytoplasmas, and others) in the header of each sequence to facilitate downstream processing. These organism categories were chosen because they include the most important plant pathogens. The plants category was defined as each sequence which had in its SILVA taxonomy the (partial) word “Plant”, “Chloroplast”, or “Mitochondr”; the spiders and mites category was assigned when the taxonomy contained the word “Arachnida”; and the oomycetes category was defined as the class Peronosporomycetes. The other categories were straightforward to extract from the SILVA taxonomy. In addition, for each sequence in the SILVA LSU database, the accession number was extracted, and the corresponding NCBI Taxonomy identifier and its complete taxonomy was retrieved using NCBI’s E-utilities. The combined accession number, organism category, and NCBI extracted taxonomy were used as a sequence header for the final database. With the database ready, a detailed tutorial was written explaining how to participate to the community effort, including tutorials on how to perform the mapping using different types of software, such as BWA [50], CLC Genomics Workbench (https://digitalinsights.qiagen.com, accessed on 26 March 2020), and Geneious (https://www.geneious.com). These are available on https://gitlab.com/ilvo/PHBN-WP4-RNAseq_Community_Screening. Invitations to participate were sent to all official and associated partners of the Euphresco “Plant Health Bioinformatics Network”, a network of researchers working with HTS data applied to plant pathogen diagnostics. Further distribution to participate among colleagues was encouraged, and an advertisement was made on social media (Twitter). Participants were required to complete a simple metadata file with some basic information on their samples (host plant, host tissue, rRNA removal step (yes/no), read length, number of reads, number of mapped reads) and upload the results of their mappings. The latter was supplied in a textual format which specifically included the number of mapped reads corresponding to each sequence of the database. This could be easily exported from graphical software (Geneious, CLC Genomics Workbench), or from command line tools using the “idxstats” program of SAMtools [51]. Finally, the results of each participant were combined in one table and unrealistic values due to errors in reporting were removed (for example, more mapped reads than input reads). Results were summarized as bar plots representing the percentage of mapped reads for each organism category (plants, bacteria, fungi, oomycetes, phytoplasmas, insects, spiders and mites, and others) using the ggplot2 package v.3.3.2 [52] in R v.4.0.3 [53] with RStudio v.1.3.1093 [54].

#### 4.2.2. Phase II: In Depth Analysis

In Phase II, the results of Phase I were used to select samples for more detailed analysis. The selection was performed quite arbitrarily, based on at least one of the following criteria: relatively high number of non-viral, non-plant reads; at least one potential plant pathogen or pest present among the most prevalent rRNA hits per organism category; or overall high biodiversity of the sample. Typically, we aimed for keeping more or less half of the datasets, ideally retaining at least one dataset per participant. Next, participants were invited to transfer the raw data of the selected samples to ILVOThe raw data of each sample was further processed by a bioinformatics expertand checked for presence of adapters using FastQC (https://www.bioinformatics.babraham.ac.uk/projects/fastqc/, accessed on 2 May 2020). If present, adapters were removed using cutadapt [45]. Next, direct taxonomic classification of the reads was performed with the k-mer based Kraken2 software [46], using the lowest common ancestor method (LCA) for assignments at different taxonomic levels. For the database for the Kraken2 analysis, a local version of the complete Genbank non-redundant Nucleotide database converted to a Kraken2 index was used [47]. The resulting taxonomic classifications were visualized using Krona [48]. All detailed analyses of selected samples are made available through reports per participant on https://gitlab.com/ilvo/PHBN-WP4-RNAseq_Community_Screening. This repository was also submitted to Zenodo under DOI 10.5281/zenodo.7974743.

#### 4.2.3. Phase III: Feedback and Confirmation

In Phase III, the participants received a report on the detailed analyses including the Krona plots (to interactively check the detailed taxonomic classification results of their samples in a web browser) and some suggestions of pathogens or pests that might be present in their dataset(s). Participants were asked to carefully review the results and give feedback on which observed non-viral pathogens or pests could potentially be present, taking into account host plant species, symptoms, sampling location, etc. Next, based on the feedback from the participants, we set a general cut-off value to classify the presence of non-viral pathogens per sample. For 7 samples for which material was still available, specific molecular confirmation tests, as described in literature, were conducted to confirm the presence of *Pectobacterium* sp. [55], ‘*Candidatus* Phytoplasma pruni’ [56], *Botrytis cinerea* [57], and/or *Alternaria* sp. [58]. This was performed on DNA extracted from the same samples for which the RNA-seq dataset was available. Furthermore, participants were also asked to complete an online questionnaire where their opinion on the usefulness of the analysis was evaluated, whether they were aware of such type of analyses, etc. The questions of the questionnaire can be found in File S2. Overall results were summarized in tables and downstream statistical tests and visualizations were performed in R v.4.0.3 [53] with RStudio v.1.3.1093 [54] using dplyr v.1.0.2 [59] and ggplot2 v.3.3.3 [52].

## Figures and Tables

**Table 1:** Overview of samples with confirmed pathogen infection used for RNA-seq analysis and of datasets downloaded from public sequence databases. The number of reads per million (rpm) together with the most likely species assignment based on the Kraken2 analysis is given. The latter was based on the species that had the majority of the reads assigned to them. In case there were multiple related species with a similar number of assigned reads, the taxon is reported at genus level. Likewise, in case of a low relative occurrence of the species (<20 rpm), the taxon is reported at genus level to avoid species assignments supported by a small number of reads.

Sample ID/Accession	Number of Reads	Host Plant	Tissue	Confirmed Pathogen Infection	Taxon Observed in Kraken2	Kraken2 rpm for Relevant Taxon
ILVO_Cpnnyn	5,274,766 (2 × 150 bp)	*Chrysanthemum* × *morifolium* (florist’s daisy)	Leaves	*Puccinia horiana*	*Puccinia graminis*	257
ILVO_Salix	5,774,434 (2 × 150 bp)	*Salix alba* (white willow)	Leaves	*Xylella fastidiosa*	*Xylella taiwanensis*	34
ILVO_Daucu	5,169,125 (2 × 150 bp)	*Daucus carota* (carrot)	Leaves	‘*Ca.* Phytoplasma asteris’	‘*Ca.* Phytoplasma asteris’	877
AGS_feve	6,680,182 (2 × 150 bp)	*Vicia faba* (broad bean)	Leaves	‘*Ca.* Phytoplasma sp.’ Flavescence dorée	‘*Ca.* Phytoplasma sp.’	339
AGS_vigne	4,051,638 (2 × 150 bp)	*Vitis vinifera* (grapevine)	Leaves	downy mildew	*Plasmopara viticola*	51,974
KIS_V3417	6,592,979 (2 × 150 bp)	*Daucus carota* (carrot)	Leaves	*Alternaria* sp.	*Alternaria solani*	11,805
KIS_V3418	7,310,393 (2 × 150 bp)	*Solanum tuberosum* (potato)	Leaves	*Alternaria* sp.	*Alternaria solani*	1191
KIS_V3408	5,891,895 (2 × 150 bp)	*Solanum lycopersicum* (tomato)	Leaves	*Phytophthora infestans*	*Phytophthora* sp.	4831
KIS_V3408dup	8,616,508 (2 × 150 bp)	*Solanum lycopersicum* (tomato)	Leaves	*Phytophthora infestans*	*Phytophthora infestans*	5288
SRR5100668	26,727,692 (1 × 150 bp)	*Solanum tuberosum* (potato)	Leaves	‘*Ca.* Liberibacter solanacearum’	‘*Ca.* Liberibacter sp.’	1
SRR10148792	17,431,858 (2 × 100 bp)	*Citrus sinensis* (orange)	Leaves	‘*Ca.* Liberibacter asiaticus’	‘*Ca.* Liberibacterasiaticus’	35
SRR8295844	16,912,818 (1 × 75 bp)	*Citrus sinensis* (orange)	Phloem	‘*Ca.* Liberibacter asiaticus’	‘*Ca.* Liberibacter asiaticus’	292
SRR9225242	98,809,288 (1 × 75 bp)	*Solanum lycopersicum* (tomato)	Leaves	‘*Ca.* Phytoplasma solani’	‘*Ca.* Phytoplasma solani’	8082
SRR7186379	61,339,166 (2 × 100 bp)	*Glycine max* (soybean)	Flower buds	‘*Ca.* Phytoplasma sp.’	‘*Ca.* Phytoplasma sp.’	1055
SRR17253894	43,453,018 (2 × 150 bp)	*Prunus pseudocerasus* (Chinese cherry)	Fruits	‘*Ca.* Phytoplasma sp.’	‘*Ca.* Phytoplasma sp.’	26
SRR8003868	50,653,038 (2 × 150 bp)	*Solanum sisymbriifolium* (sticky nightshade)	Roots	*Verticillium dahliae*	*Verticillium dahliae*	12,784
SRR6760520	88,633,716 (2 × 100 bp)	*Olea europaea* (olive)	Roots	*Verticillium dahliae*	*Verticillium dahliae*	34,880
SRR1525437	73,799,968 (2 × 100 bp)	*Olea europaea* (olive)	Leaves	*Verticillium dahliae*	*Verticillium dahliae*	40
SRR6053344	61,546,620 (2 × 125 bp)	*Gossypium hirsutum* (upland cotton)	Roots	*Verticillium dahliae*	*Verticillium dahliae*	12,610
SRR7814393	11,481,479 (1 × 100 bp)	*Malus domestica* (apple)	Leaves	*Erwinia amylovora*	*Erwinia* sp.	4
SRR13488408	46,302,968 (2 × 150 bp)	*Capsicum annuum* (pepper)	Leaves	*Xanthomonas campestris* pv. vesicatoria	*Xanthomonas* sp.	13
ERR2036424	38,196,926 (1 × 100 bp)	*Triticum aestivum* (wheat)	Roots	*Xanthomonas translucens*	*Xanthomonas* sp.	4

**Table 2:** Overview of the 37 selected datasets used in the detailed analysis showing the Sample ID, host plant, tissue, and observed pathogens/pests. Pathogens/pests shown in the table have ≥100 rpm in the datasets according to the Kraken2 analysis, as mentioned between brackets.

Sample ID	Host Plant	Tissue	Pathogens/Pests as Identified by Kraken2 (rpm)	Pathogen/Pest Group(s)
Agroscope_OS_Solanum_S1	*Solanum tuberosum* (potato)	tubers	*Rhizoctonia solani* (1494), *Fusarium* sp. (430), *Colletotrichum* sp. (238)	fungi
BAEM_Vitis_S1	*Vitis vinifera* (grapevine)	leaves	*Alternaria* sp. (996), *Puccinia* sp. (565), *Drosophila* sp. (305), *Agrobacterium tumefaciens* (now *Rhizobium vitis*) (155)	fungi, insects, bacteria
ILVO_Capsicum_S11	*Capsicuum annuum* (sweet pepper)	leaves	*Acyrthosiphon pisum* (835), *Thrips tabaci* (476), *Tetranychidae* (430), *Myzus persicae* (216), *Frankliniella occidentalis* (203)	insects, mites
ILVO_Capsicum_S8	*Capsicuum annuum* (pepper)	leaves	*Myzus persicae* (87,467), *Acyrthosiphon pisum* (32,131), *Alternaria* sp. (127)	insects, fungi
ILVO_Citrus_S2	*Citrus sinensis* (orange)	leaves	*Fusarium* sp. (205), *Alternaria* sp. (169), *Botrytis cinerea* (131)	fungi
ILVO_Ipomea_S13	*Ipomea batatas* (sweet potato)	tubers	*Frankliniella occidentalis* (667), *Fusarium* sp. (581), *Botrytis cinerea* (201)	insects, fungi
ILVO_Ipomea_S4	*Ipomea batatas* (sweet potato)	leaves	*Alternaria* sp. (261)	fungi
ILVO_Oxalis_S14	*Oxalis tuberosa* (oca)	tubers	*Drosophila* sp. (566), *Aphis gossypii* (320), *Tetranychus urticae* (105)	insects, mites
ILVO_Solanum_S10	*Solanum lycopersicum* (tomato)	leaves	Eriophyidae (2760), Erysiphaceae (443)	mites, fungi
ILVO_Solanum_S5	*Solanum lycopersicum* (tomato)	leaves	*Drosophila melanogaster* (2808)	insects
ILVO_Solanum_S7	*Solanum lycopersicum* (tomato)	leaves	*Botrytis cinerea* (6442), *Passalora fulva* (446), *Erysiphaceae* (322)	fungi
ILVO_Solanum_S9	*Solanum melongena* (eggplant)	leaves	*Myzus persicae* (104,861), *Acyrthosiphon pisum* (23,004), *Albugo* sp. (1086), *Botrytis cinerea* (923), *Alternaria* sp. (119)	insects, oomycetes, fungi
ILVO_Stachys_S1	*Stachys affinis* (crosne)	leaves	*Alternaria* sp. (317), *Tetranychus urticae* (760), *Frankliniella occidentalis* (186), *Botrytis cinerea* (131)	insects, mites, fungi
INRAE_Vitis_S2	*Vitis vinifera* (grapevine)	rootstock	*Botrytis cinerea* (84,898)	fungi
INRAE_Vitis_S3	*Vitis vinifera* (grapevine)	rootstock	*Botrytis cinerea* (24,342)	fungi
JKI_Beta_S1	*Beta vulgaris* subsp. *Vulgaris* (beet)	leaves	*Pantoea agglomerans* (12,325), *Alternaria* sp. (907), *Fusarium* sp. (679), *Verticillium* sp. (170), *Stemphylium* sp. (101)	bacteria, fungi
KIS_Allium_S6	*Allium sativum* (garlic)	leaves	*Fusarium* sp. (726)	fungi
KIS_Glycine_S1	*Glycine max* (soybean)	leaves	*Phytophthora* sp. (276)	oomycetes
KIS_Glycine_S2	*Glycine max* (soybean)	leaves	*Tetranychus urticae* (345), *Phytophthora* sp. (295)	mites, oomycetes
KIS_Rubus_S8	*Rubus* sp. (blackberry)	leaves	*Aphis gossypii* (3063)	insects
NAK_Solanum_S1	*Solanum tuberosum* (potato)	tubers	*Rhizoctonia solani* (352), *Pectobacterium* sp. (145)	fungi, bacteria
NCL_Poaceae-pool_S1	Pool of Apiaceae	leaves	*Albugo* sp. (107,450), *Erysiphaceae* (11,581), *Acyrthosiphon pisum* (588), *Alternaria* sp. (267), *Puccinia* sp. (110)	oomycetes, fungi, insects
UCDavis_Fragaria_S13	*Fragaria* × *ananassa* (strawberry)	leaves and petioles	*Frankliniella occidentalis* (313)	insects
UCDavis_Prunus_S7	*Prunus cerasus* (cherry)	petioles	‘*Ca.* Phytoplasma pruni’ (310)	phytoplasma
UCDavis_Rosa_S10	*Rosa* sp. (rose)	leaves and petioles	*Tetranychus urticae* (2728)	mites
UCDavis_Rosa_S11	*Rosa* sp. (rose)	leaves and petioles	*Drosophila* sp. (165)	insects
ULG_Musa_S2	*Musa* sp. (banana)	leaves	*Tetranychus urticae* (100)	mites
USDA_Pyrus_S6	*Pyrus communis* (pear)	leaves	*Tetranychus urticae* (1680), Pseudococcidae (351)	mites, insects
USDA_Pyrus_S7	*Pyrus communis* (pear)	leaves	*Tetranychus urticae* (4244)	mites
INRAE_Prunus_S4	*Prunus domestica* (plum)	leaves	none	
MPI_CP_Macropiper_S5	*Macropiper excelsum* (kawakawa)	leaves	none	
MPI_CP_Melicytus_S3	*Melicytus* sp.	leaves	none	
MPI_DW_Allium_S2	*Allium triquetrum* (onion weed)	leaves	none	
MPI_DW_Clematis_S3	*Clematis paniculata*	leaves	none	
MPI_DW_Melicytus_S7	*Melicytus ramiflorus*	leaves	none	
NIB_Pool_S1	Pool of plants from Solanaceae family	leaves	none	
USDA_Saccharum_S8	*Saccharum* sp. (sugarcane)	leaves	none	

**Table 3:** Overview of confirmation assays. The sample ID and host plant are shown, together with the tested pathogen, the observed reads in the Kraken2 analysis (in rpm), and the result of the molecular test.

Sample ID	Sample Origin	Pathogen	Kraken2 (rpm)	(q)PCR Result
NAK_Solanum_S1	Potato tuber (pool of 12 samples)	*Pectobacterium* sp.	145	Positive for *P. brasiliense* in 9/12 individual samples (Ct range: 29.9–38.7)
UCDAVIS_Prunus_S7	Cherry leaf	‘*Ca.* Phytoplasma pruni’	310	Positive (Ct = 19.189)
INRAE_Vitis_S3	Grapevine rootstock	*Botrytis cinerea*	24,342	Positive
ILVO_Solanum_S7	Tomato leaf	*Botrytis cinerea*	6442	Positive
ILVO_Stachys_S1	Crosne leaf	*Botrytis cinerea*	131	Negative
ILVO_Capsicum_S8	Pepper leaf	*Botrytis cinerea*	78	Negative
ILVO_Solanum_S10	Tomato leaf	*Botrytis cinerea*	51	Negative
ILVO_Stachys_S1	Crosne leaf	*Alternaria* sp.	1366	Positive
ILVO_Capsicum_S8	Pepper leaf	*Alternaria* sp.	127	Positive
ILVO_Solanum_S7	Tomato leaf	*Alternaria* sp.	30	Positive
ILVO_Solanum_S10	Tomato leaf	*Alternaria* sp.	17	Negative

## Data Availability

The results of the RNA-seq community effort can be checked and downloaded from https://gitlab.com/ilvo/PHBN-WP4-RNAseq_Community_Screening. This repository was also submitted to Zenodo under DOI 10.5281/zenodo.7974743. Raw sequencing data generated during this study was submitted to the NCBI Sequence Read Archive (SRA) under BioProject number PRJNA961939.

## Supplementary Materials

The following supporting information can be downloaded at: https://www.mdpi.com/2223-7747/12/11/2139/s1, Figure S1: Overview of the datasets per participant (101 in total) that entered the “RNA-seq community effort”; File S1: Overview of the participants and datasets; File S2: Questionnaire sent to the participants at the time they received the results of the detailed analysis of their sample(s); File S3: Answers from participants to the questionnaire.

## References

[1] Handelsman J., Rondon M.R., Brady S.F., Clardy J., Goodman R.M. (1998). Molecular biological access to the chemistry of unknown soil microbes: A new frontier for natural products. Chem. Biol..

[2] Goldberg B., Sichtig H., Geyer C., Ledeboer N., Weinstock G.M. (2015). Making the leap from research laboratory to clinic: Challenges and opportunities for next-generation sequencing in infectious disease diagnostics. MBio.

[3] Forbes J.D., Knox N.C., Ronholm J., Pagotto F., Reimer A. (2017). Metagenomics: The next culture-independent game changer. Front. Microbiol..

[4] Lebas B., Adams I., Al Rwahnih M., Baeyen S., Bilodeau G.J., Blouin A.G., Boonham N., Candresse T., Chandelier A., De Jonghe K. (2022). Facilitating the adoption of high-throughput sequencing technologies as a plant pest diagnostic test in laboratories: A step-by-step description. EPPO Bull..

[5] Hardwick S., Deveson I.W., Mercer T.R. (2017). Reference standards for next-generation sequencing. Nat. Rev. Genet..

[6] Chiu C.Y., Miller S.A. (2019). Clinical metagenomics. Nat. Rev. Genet..

[7] Kreuze J.F., Perez A., Untiveros M., Quispe D., Fuentes S., Barker I., Simon R. (2009). Complete viral genome sequence and discovery of novel viruses by deep sequencing of small RNAs: A generic method for diagnosis, discovery and sequencing of viruses. Virology.

[8] Adams I.P., Glover R.H., Monger W.A., Mumford R., Jackeviciene E., Navalinskiene M., Samuitiene M., Boonham N. (2009). Next-generation sequencing and metagenomic analysis: A universal diagnostic tool in plant virology. Mol. Plant Pathol..

[9] Al Rwahnih M., Daubert S., Golino D., Rowhani A. (2009). Deep sequencing analysis of RNAs from a grapevine showing Syrah decline symptoms reveals a multiple virus infection that includes a novel virus. Virology.

[10] Donaire L., Wang Y., Gonzalez-Ibeas D., Mayer K.F., Aranda M.A., Llave C. (2009). Deep-sequencing of plant viral small RNAs reveals effective and widespread targeting of viral genomes. Virology.

[11] Massart S., Olmos A., Jijakli H., Candresse T. (2014). Current impact and future directions of high throughput sequencing in plant virus diagnostics. Virus Res..

[12] Pecman A., Kutnjak D., Gutiérrez-Aguirre I., Adams I., Fox A., Boonham N., Ravnikar M. (2017). Next generation sequencing for detection and discovery of plant viruses and viroids: Comparison of two approaches. Front. Microbiol..

[13] Adams I., Fox A., Wang A., Zhou X. (2016). Diagnosis of plant viruses using next-generation sequencing and metagenomic analysis. Current Research Topics in Plant Virology.

[14] Maree H.J., Fox A., Al Rwahnih M., Boonham N. (2018). Application of HTS for routine plant virus diagnostics: State of the art and challenges. Front. Plant Sci..

[15] Tedersoo L., Drenkhan R., Anslan S., Morales-Rodriguez C., Cleary M. (2019). High-throughput identification and diagnostics of pathogens and pests: Overview and practical recommendations. Mol. Ecol. Resour..

[16] Badial A.B., Sherman D., Stone A., Gopakumar A., Wilson V., Schneider W., King J. (2018). Nanopore sequencing as a surveillance tool for plant pathogens in plant and insect tissues. Plant Dis..

[17] Chalupowicz L., Dombrovsky A., Gaba V., Luria N., Reuven M., Beerman A., Lachman O., Dror O., Nissan G., Manulis-Sasson S. (2019). Diagnosis of plant diseases using the Nanopore sequencing platform. Plant Pathol..

[18] Espindola A., Schneider W., Hoyt P.R., Marek S.M., Garzon C. (2015). A new approach for detecting fungal and oomycete plant pathogens in next generation sequencing metagenome data utilising electronic probes. Int. J. Data Min. Bioinform..

[19] Stobbe A.H., Daniels J., Espindola A.S., Verma R., Melcher U., Ochoa-Corona F., Garzon C., Fletcher J., Schneider W. (2013). E-probe Diagnostic Nucleic acid Analysis (EDNA): A theoretical approach for handling of next generation sequencing data for diagnostics. J. Microbiol. Methods.

[20] Gómez-Rodríguez C., Timmermans M.J.T.N., Crampton-Platt A., Vogler A.P. (2017). Intraspecific genetic variation in complex assemblages from mitochondrial metagenomics: Comparison with DNA barcodes. Methods Ecol. Evol..

[21] Adams I.P., Fox A., Boonham N., Massart S., De Jonghe K. (2018). The impact of high throughput sequencing on plant health diagnostics. Eur. J. Plant Pathol..

[22] Gaafar Y.Z.A., Ziebell H. (2020). Comparative study on three viral enrichment approaches based on RNA extraction for plant virus/viroid detection using high-throughput sequencing. PLoS ONE.

[23] Kutnjak D., Tamisier L., Adams I., Boonham N., Candresse T., Chiumenti M., De Jonghe K., Kreuze J.F., Lefebvre M., Silva G. (2021). A primer on the analysis of high-throughput sequencing data for detection of plant viruses. Microorganisms.

[24] Chandler J.A., Liu R.M., Bennett S.N. (2015). RNA Shotgun metagenomic sequencing of northern California (USA) mosquitoes uncovers viruses, bacteria, and fungi. Front. Microbiol..

[25] Toffolatti S.L., De Lorenzis G., Brilli M., Moser M., Shariati V., Tavakol E., Maddalena G., Passera A., Casati P., Pindo M. (2020). Novel aspects on the interaction between grapevine and *Plasmopara viticola*: Dual-RNA-seq analysis highlights gene expression dynamics in the pathogen and the plant during the battle for infection. Genes.

[26] Kawahara Y., Oono Y., Kanamori H., Matsumoto T., Itoh T., Minami E. (2012). Simultaneous RNA-seq analysis of a mixed transcriptome of rice and blast fungus interaction. PLoS ONE.

[27] Musungu B., Bhatnagar D., Quiniou S., Brown R.L., Payne G.A., O’Brian G., Fakhoury A.M., Geisler M. (2020). Use of Dual RNA-seq for systems biology analysis of *Zea mays* and *Aspergillus flavus* interaction. Front. Microbiol..

[28] Nerva L., Garcia J.F., Favaretto F., Giudice G., Moffa L., Sandrini M., Cantu D., Zanzotto A., Gardiman M., Velasco R. (2022). The hidden world within plants: Metatranscriptomics unveils the complexity of wood microbiomes. J. Exp. Bot..

[29] Law S.R., Serrano A.R., Daguerre Y., Sundh J., Schneider A.N., Stangl Z.R., Castro D., Grabherr M., Näsholm T., Street N.R. (2022). Metatranscriptomics captures dynamic shifts in mycorrhizal coordination in boreal forests. Proc. Natl. Acad. Sci. USA.

[30] Llontop M.E.M., Sharma P., Flores M.A., Yang S., Pollok J., Tian L., Huang C., Rideout S., Heath L.S., Li S. (2020). Strain-level identification of bacterial tomato pathogens directly from metagenomic sequences. Phytopathology.

[31] Xu R., Adam L., Chapados J., Soliman A., Daayf F., Tambong J.T. (2021). MinION Nanopore-based detection of *Clavibacter nebraskensis*, the corn Goss’s wilt pathogen, and bacteriomic profiling of necrotic lesions of naturally-infected leaf samples. PLoS ONE.

[32] Hu Y., Green G.S., Milgate A.W., Stone E.A., Rathjen J.P., Schwessinger B. (2019). Pathogen detection and microbiome analysis of infected wheat using a portable DNA sequencer. Phytobiomes J..

[33] Loit K., Adamson K., Bahram M., Puusepp R., Anslan S., Kiiker R., Drenkhan R., Tedersood L. (2019). Relative performance of MinION (Oxford Nanopore Technologies) versus Sequel (Pacific Biosciences) third-generation sequencing instruments in identification of agricultural and forest fungal pathogens. Appl. Environ. Microbiol..

[34] Yang S., Johnson M.A., Hansen M.A., Bush E., Li S., Vinatzer B.A. (2022). Metagenomic sequencing for detection and identification of the boxwood blight pathogen *Calonectria pseudonaviculata*. Sci. Rep..

[35] Johnson M.A., Liu H., Bush E., Sharma P., Yang S., Mazloom R., Heath L.S., Nita M., Li S., Vinatzer B.A. (2022). Investigating plant disease outbreaks with long-read metagenomics: Sensitive detection and highly resolved phylogenetic reconstruction applied to *Xylella fastidiosa*. Microb. Genomics.

[36] Kulik T., Molcan T., Fiedorowicz G., van Diepeningen A., Stakheev A., Treder K., Olszewski J., Bilska K., Beyer M., Pasquali M. (2022). Whole-genome single nucleotide polymorphism analysis for typing the pandemic pathogen *Fusarium graminearum* sensu stricto. Front. Microbiol..

[37] Rong W., Rollin J., Hanafi M., Roux N., Massart S. (2022). Validation of high throughput sequencing as virus indexing test for *Musa* germplasm: Performance criteria evaluation and contamination monitoring using an alien control. PhytoFrontiers.

[38] Malnoy M., Martens S., Norelli J.L., Barny M.-A., Sundin G.W., Smits T.H.M., Duffy B. (2012). Fire blight: Applied genomic insights of the pathogen and host. Annu. Rev. Phytopathol..

[39] Jacques M.A., Arlat M., Boulanger A., Boureau T., Carrère S., Cesbron S., Chen N.W.G., Cociancich S., Darrasse A., Denancé N. (2016). Using ecology, physiology, and genomics to understand host specificity in *Xanthomonas*. Annu. Rev. Phytopathol..

[40] Hu J., Wei Z., Kowalchuk G.A., Xu Y., Shen Q., Jousset A. (2020). Rhizosphere microbiome functional diversity and pathogen invasion resistance build up during plant development. Environ. Microbiol..

[41] Lazcano C., Boyd E., Holmes G., Hewavitharana S., Pasulka A., Ivors K. (2021). The rhizosphere microbiome plays a role in the resistance to soil-borne pathogens and nutrient uptake of strawberry cultivars under field conditions. Sci. Rep..

[42] Ye S.H., Siddle K.J., Park D.J., Sabeti P.C. (2019). Benchmarking metagenomics tools for taxonomic classification. Cell.

[43] Kalantar K.L., Carvalho T., De Bourcy C.F.A., Dimitrov B., Dingle G., Egger R., Han J., Holmes O.B., Juan Y.F., King R. (2021). IDseq—An open source cloud-based pipeline and analysis service for metagenomic pathogen detection and monitoring. Gigascience.

[44] Menzel P., Ng K.L., Krogh A. (2016). Fast and sensitive taxonomic classification for metagenomics with Kaiju. Nat. Commun..

[45] Martin M. (2011). Cutadapt removes adapter sequences from high-throughput sequencing reads. EMBnet. J..

[46] Wood D.E., Lu J., Langmead B. (2019). Improved metagenomic analysis with Kraken 2. Genome Biol..

[47] Sayers E.W., Beck J., Brister J.R., Bolton E.E., Canese K., Comeau D.C., Funk K., Ketter A., Kim S., Kimchi A. (2020). Database resources of the National Center for Biotechnology Information. Nucleic Acids Res..

[48] Ondov B.D., Bergman N.H., Phillippy A.M. (2011). Interactive metagenomic visualization in a web browser. BMC Bioinform..

[49] Yilmaz P., Parfrey L.W., Yarza P., Gerken J., Pruesse E., Quast C., Schweer T., Peplies J., Ludwig W., Glöckner F.O. (2014). The SILVA and “all-species Living Tree Project (LTP)” taxonomic frameworks. Nucleic Acids Res..

[50] Li H., Durbin R. (2009). Fast and accurate short read alignment with Burrows-Wheeler transform. Bioinformatics.

[51] Li H., Handsaker B., Wysoker A., Fennell T., Ruan J., Homer N., Marth G., Abecasis G., Durbin R. (2009). The Sequence Alignment/Map format and SAMtools. Bioinformatics.

[52] Wickham H. (2016). ggplot2 Elegant Graphics for Data Analysis.

[53] R Core Team R Core Team (2020). R: A Language and Environment for Statistical Computing.

[54] RStudio Team (2020). RStudio: Integrated Development for R.

[55] Muzhinji N., Dube J., de Haan E., Woodhall J., van der Waals J. (2020). Development of a TaqMan PCR assay for specific detection and quantification of *Pectobacterium brasiliense* in potato tubers and soil. Eur. J. Plant Pathol..

[56] Hodgetts J., Boonham N., Mumford R., Dickinson M. (2009). Panel of 23S rRNA gene-based real-time PCR assays for improved universal and group-specific detection of phytoplasmas. Appl. Environ. Microbiol..

[57] Fan X., Zhang J., Yang L., Wu M., Chen W., Li G. (2015). Development of PCR-based assays for detecting and differentiating three species of *Botrytis* infecting broad bean. Plant Dis..

[58] Chakdar H., Goswami S.K., Singh E., Choudhary P., Yadav J., Kashyap P.L., Srivastava A.K., Saxena A.K. (2019). noxB-based marker for *Alternaria* spp.: A new diagnostic marker for specific and early detection in crop plants. 3 Biotech.

[59] Wickham H., Francois R., Henry L., Müller K. (2020). dplyr: A Grammar of Data Manipulation. R Packag. Version 1.0.2. https://dplyr.tidyverse.org/.

