# Correction: Haegeman et al. Looking beyond Virus Detection in RNA Sequencing Data: Lessons Learned from a Community-Based Effort to Detect Cellular Plant Pathogens and Pests. *Plants* 2023, *12*, 2139

**DOI:** 10.3390/plants13050623

**Published:** 2024-02-24

**Authors:** Annelies Haegeman, Yoika Foucart, Kris De Jonghe, Thomas Goedefroit, Maher Al Rwahnih, Neil Boonham, Thierry Candresse, Yahya Z. A. Gaafar, Oscar P. Hurtado-Gonzales, Zala Kogej Zwitter, Denis Kutnjak, Janja Lamovšek, Marie Lefebvre, Martha Malapi, Irena Mavrič Pleško, Serkan Önder, Jean-Sébastien Reynard, Ferran Salavert Pamblanco, Olivier Schumpp, Kristian Stevens, Chandan Pal, Lucie Tamisier, Çiğdem Ulubaş Serçe, Inge van Duivenbode, David W. Waite, Xiaojun Hu, Heiko Ziebell, Sébastien Massart

**Affiliations:** 1 Plant Sciences Unit, Flanders Research Institute for Agriculture, Fisheries and Food (ILVO), 9820 Merelbeke, Belgium; yoika.foucart@ilvo.vlaanderen.be (Y.F.); kris.dejonghe@ilvo.vlaanderen.be (K.D.J.); thomas.goedefroit@ilvo.vlaanderen.be (T.G.); 2 Foundation Plant Services, Department of Plant Pathology, University of California, Davis, CA 95616, USA; malrwahnih@ucdavis.edu (M.A.R.); kastevens@ucdavis.edu (K.S.); 3 School of Natural and Environmental Sciences, Newcastle University, Newcastle Upon Tyne NE1 7RU, UK; neil.boonham@newcastle.ac.uk (N.B.); f.salavert2@newcastle.ac.uk (F.S.P.); 4 UMR 1332 Biologie du Fruit et Pathologie, Institut National de Recherche pour l’Agriculture, l’Alimentation et l’Environnement (INRAE), Université de Bordeaux, 33882 Villenave-d’Ornon, France; thierry.candresse@inrae.fr (T.C.); marie.lefebvre@inrae.fr (M.L.); 5 Centre for Plant Health, Canadian Food Inspection Agency, 8801 East Saanich Road, North Saanich, BC V8L 1H3, Canada; yahya.gaafar@inspection.gc.ca; 6 Plant Germplasm Quarantine Program, Animal and Plant Health Inspection Service, United States Department of Agriculture (USDA-APHIS), Beltsville, ML 20705, USA; oscar.hurtado-gonzales@usda.gov (O.P.H.-G.); xiaojun.hu@usda.gov (X.H.); 7 Department of Biotechnology and Systems Biology, National Institute of Biology (NIB), 1000 Ljubljana, Slovenia; zala.kogej@nib.si (Z.K.Z.); denis.kutnjak@nib.si (D.K.); 8 Jožef Stefan International Postgraduate School, 1000 Ljubljana, Slovenia; 9 Plant Protection Department, Agricultural Institute of Slovenia (KIS), 1000 Ljubljana, Slovenia; janja.lamovsek@kis.si (J.L.); irena.mavricplesko@kis.si (I.M.P.); 10 Biotechnology Risk Analysis Program, Animal and Plant Health Inspection Service, United States Department of Agriculture (USDA-APHIS), Riverdale, ML 20737, USA; martha.malapi@usda.gov; 11 Department of Plant Protection, Faculty of Agriculture, Eskişehir Osmangazi University, Odunpazarı, Eskişehir 26160, Turkey; onderserkan@gmail.com; 12 Department of Plant Protection, Agroscope, 1260 Nyon, Switzerland; jean-sebastien.reynard@agroscope.admin.ch (J.-S.R.); olivier.schumpp@agroscope.admin.ch (O.S.); 13 Zespri International Limited, 400 Maunganui Road, Mount Maunganui 3116, New Zealand; chandan_pal143@yahoo.com; 14 Unités GAFL et Pathologie Végétale, Institut National de Recherche pour l’Agriculture, l’Alimentation et l’Environnement (INRAE), 84143 Montfavet, France; lucie.tamisier@inrae.fr; 15 Department of Plant Production and Technologies, Faculty of Agricultural Sciences and Technologies, Niğde Ömer Halisdemir University, Niğde 51240, Turkey; culubas@gmail.com; 16 Dutch General Inspection Service for Agricultural Seed and Seed Potatoes (NAK), Randweg 14, 8304 AS Emmeloord, The Netherlands; i.vanduivenbode@nak.nl; 17 Plant Health and Environment Laboratory, Ministry for Primary Industries, Auckland 1140, New Zealand; david.waite@mpi.govt.nz; 18 Institute for Epidemiology and Pathogen Diagnostics, Federal Research Centre for Cultivated Plants, Julius Kühn Institute (JKI), Messeweg 11-12, 38104 Braunschweig, Germany; heiko.ziebell@julius-kuehn.de; 19 Plant Pathology Laboratory, University of Liège, Gembloux Agro-Bio Tech, TERRA, 5030 Gembloux, Belgium

In the original publication [1], there were two mistakes in Figure 3 as published. The legend showed the wrong descriptions with the colors, and the Y-axis scale was not correctly labeled. The corrected Figure 3 with an adjusted description appears below.

The authors state that the scientific conclusions are unaffected. This correction was approved by the Academic Editor. The original publication has also been updated.

## Figures and Tables

**Figure 3:**
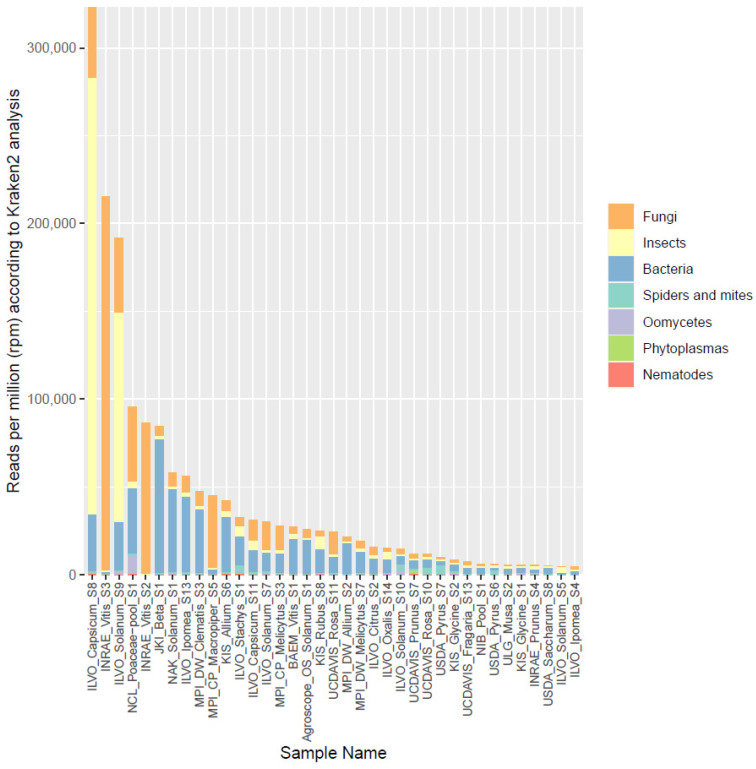
Overview of the proportion of reads (in reads per million) assigned by Kraken2 to different broad organismal categories (fungi, insects, bacteria, spiders and mites, oomycetes, phytoplasmas, and nematodes) for the 37 selected datasets.
