# Supplementary material for: Looking beyond Virus Detection in RNA Sequencing Data: Lessons Learned from a Community-Based Effort to Detect Cellular Plant Pathogens and Pests
Source: Plants (Basel). 2023 May 29;12(11):2139. doi: 10.3390/plants12112139 (PMC10255714; doi:10.3390/plants12112139)
Supplement: Supplementary file 1 [file plants-12-02139-s001.zip › Supplementary File S2 Questionnaire.pdf]

Supplementary File S2. Questionnaire sent to the participants at the time they received the results of the detailed analysis of their sample(s)

Indicate the type of lab you work in.

- ☐ Research
- ☐ Diagnostics
- ☐ Both research and diagnostics
- ☐ Other:

How would you describe your experience with high throughput sequencing data analysis?

- ☐ Beginner
- ☐ Intermediate
- ☐ Expert
- ☐ Other:

Were you already aware of the existence of the non-viral plant pathogens found in you sample(s)?

- ☐ Yes
- ☐ No
- ☐ Not applicable, since no non-viral pathogens seem to be present in the data.
- ☐ Other:

Were you aware of the existence of these kind of metatranscriptomics analysis techniques to taxonomically classify all sequences?

- ☐ Yes
- ☐ No

Do you already use these kind of techniques to check the presence of non-viral organisms in your data?

- ☐ Always
- ☐ Sometimes
- ☐ Never

To you opinion, what analysis seems to reflect best the true composition of the samples?

- ☐ Analysis 1: assembly, diamond blastx against Uniprot
- ☐ Analysis 2 : kraken2 read classification using Genbank nr Nucleotide
- ☐ I don't know.

In this community RNA-seq effort, did you find non-viral plant pathogens of interest which might be present in your sample(s)?

- ☐ Yes, but in most cases I was already aware of their presence.
- ☐ Yes, and in most cases I was not aware yet of their presence.
- ☐ I am not sure, I need help in the interpretation of the results.
- ☐ No
- ☐ No, but I have in other samples that did not participate in the challenge.
- ☐ Other:

If you answered "Yes" in the previous question, to which group did the pathogens belong(? (multiple answers possible)

- ☐ Insects
- ☐ Spiders/mites
- ☐ Fungi
- ☐ Oomycetes
- ☐ Bacteria
- ☐ Phytoplasmas
- ☐ Nematodes
- ☐ Other:

How likely is it that you would share/discuss these results with non-virologist colleagues (bacteriologists, mycologists, entomologists, etc.)?

	1	2	3	4	5	
unlikely	○	○	○	○	○	very likely

Did you compare the plant viruses you identified earlier (using your own pipeline) with the viruses found by the two taxonomic classification approaches?

- ☐ No
- ☐ Yes, and in most cases the viruses identified corresponded well.
- ☐ Yes, and in most cases, the analyses missed some viruses (false negatives).
- ☐ Yes, and in most cases, the analysis yielded more viruses than expected (either false positives, or viruses that were missed during my own analysis).
- ☐ Other:

How useful is it in your opinion to do this kind of analysis?

	1	2	3	4	5	
not useful	○	○	○	○	○	very useful

Do you think this type of analysis can be used to identify potential virus vectors (insects, mites, etc.)?

- ☐ Yes
- ☐ No
- ☐ Maybe
- ☐ I don’t know.

Would you consider doing this taxonomic classification analysis yourself in the future?

- ☐ Yes
- ☐ Maybe
- ☐ No
- ☐ I am already doing this type of analysis.
