# Supplementary figures and images for: Looking beyond Virus Detection in RNA Sequencing Data: Lessons Learned from a Community-Based Effort to Detect Cellular Plant Pathogens and Pests
Source: Plants (Basel). 2023 May 29;12(11):2139. doi: 10.3390/plants12112139 (PMC10255714; doi:10.3390/plants12112139)

Participant

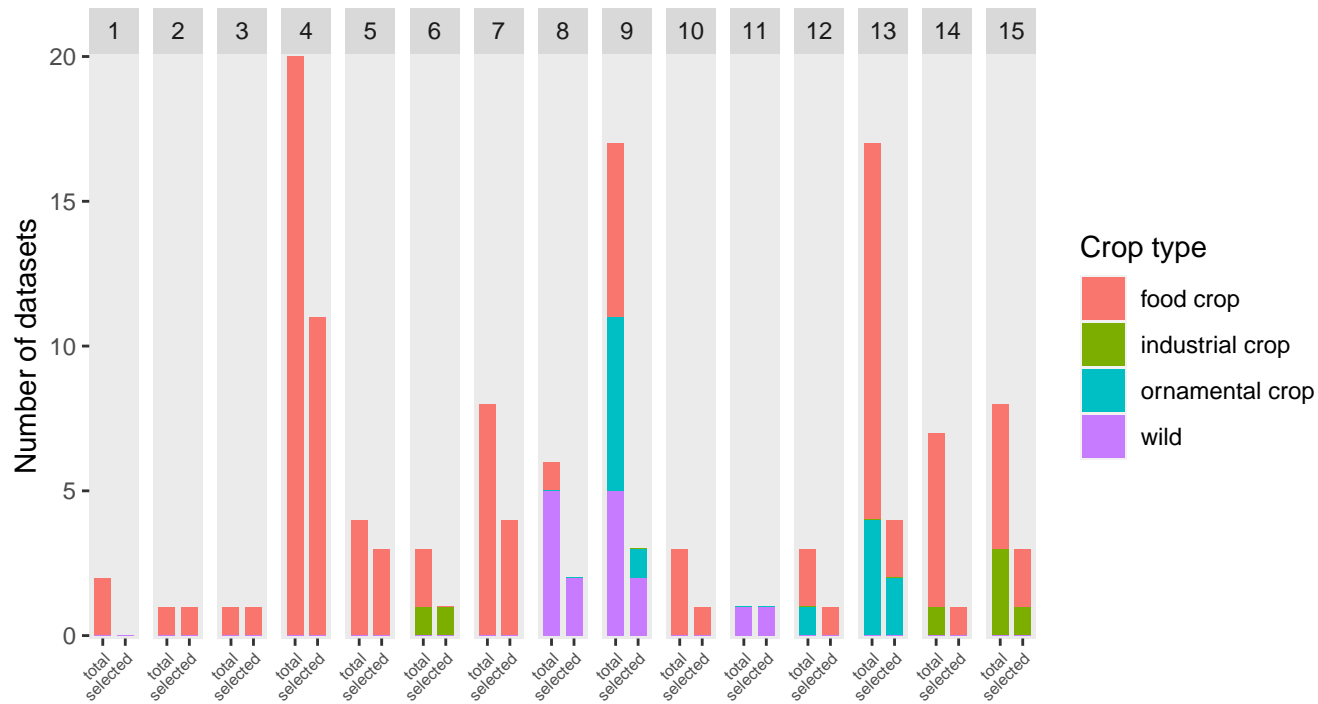

Supplement: Supplementary file 1 [file plants-12-02139-s001.zip › Figure S1 Datasets per participant that entered the RNA-seq community effort.pdf]
